# The Role of Cytochromes P450 in Infection

**DOI:** 10.3389/fimmu.2018.00089

**Published:** 2018-01-31

**Authors:** Elisavet Stavropoulou, Gratiela G. Pircalabioru, Eugenia Bezirtzoglou

**Affiliations:** ^1^School of Medicine, Democritus University of Thrace, Alexandroupolis, Greece; ^2^Research Institute of University of Bucharest, Bucharest, Romania; ^3^Department of Food Science and Technology, Faculty of Agricultural Development, Democritus University of Thrace, Laboratory of Microbiology, Biotechnology and Hygiene, Orestiada, Greece

**Keywords:** cytochromes, P450 cytochrome, microbiota and immunity, infection, inflammation

## Abstract

Cytochromes are expressed in many different tissues of the human body. They are found mostly in intestinal and hepatic tissues. Cytochromes P450 (CYPs) are enzymes that oxidize substances using iron and are able to metabolize a large variety of xenobiotic substances. CYP enzymes are linked to a wide array of reactions including and O-dealkylation, S-oxidation, epoxidation, and hydroxylation. The activity of the typical P450 cytochrome is influenced by a variety of factors, such as genus, environment, disease state, herbicide, alcohol, and herbal medications. However, diet seems to play a major role. The mechanisms of action of dietary chemicals, macro- and micronutrients on specific CYP isoenzymes have been extensively studied. Dietary modulation has effects upon the metabolism of xenobiotics. Cytochromes harbor intra- or interindividual and intra- or interethnic genetic polymorphisms. Bacteria were shown to express CYP-like genes. The tremendous metabolic activity of the microbiota is associated to its abundant pool of CYP enzymes, which catalyze phase I and II reactions in drug metabolism. Disease states, intestinal disturbances, aging, environmental toxic effects, chemical exposures or nutrition modulate the microbial metabolism of a drug before absorption. A plethora of effects exhibited by most of CYP enzymes can resemble those of proinflammatory cytokines and IFNs. Moreover, they are involved in the initiation and persistence of pathologic pain by directly activating sensory neurons and inflammatory cytokines.

## Introduction

Over centuries the human immune system has evolved to battle against pathogenic microorganisms. The ability of the immune system to generate immune responses decreases by gradual aging, due to an age-related immunodeficiency called “immunosenescence” leading to an increased susceptibility to infection in all living beings (1). Aging seems to be influenced by several factors such as genotype and lifestyle (2). Aging, immunodeficiency, and infectious disease states could modulate drug metabolism and pharmacokinetics. The major organs reported for drug clearance are the liver and the kidneys. Among these, the liver is an essential site of metabolism clearance (2). Infection and inflammation are closely connected to the hepatic and extrahepatic metabolism of cytochromes P450 (CYPs), enzymes (2).

Cytochromes are proteins belonging in superfamilies containing heme as a cofactor. Therefore, they are called hemoproteins and are used as substrates in enzymatic reactions. They are also called CYPs. The term “P450” is issued from the spectrophotometric peak obtained at the maximum optic density of the enzyme (450 nm) when it is in its reduced state associated with carbon monoxide (2).

Cytochromes P450 are the terminal oxidases in electron transfer chains reported as P450-containing systems. It must be highlighted that more than 50,000 CYP enzymes have been described in most forms of life: archaea, viruses, protists, bacteria, animals, plants, and fungi (2). It is known that CYP enzymes metabolize a wide array of xenobiotic substances. Several studies were performed to analyze their role (2), as cytochromes are located in both the liver and in the intestine. CYP enzymes seem to be influenced by infection and injury. Recent knowledge brought in light the impact of inflammatory mediators on the expression of CYP enzymes in animal models (3).

As stated previously (2, 3), expression of CYPs enzymes is mainly downregulated in the hepatic tissue during the host response to inflammation or infection, inducing changes in drug activity, and resulting in toxin release. In addition, proinflammatory cytokines such as interleukin (IL)-6, IL-1β, and tumor necrosis factor α (TNFα) are the key inflammatory mediators modulating the synthesis of acute phase proteins (APP) in inflammation. In this vein, research must be focused on the mechanism of regulation of drug metabolizing enzymes in the different disease states related to the above cytokines.

## Structure and Properties of P450

It is estimated that humans harbor 30 CYP enzymes which are involved in drug metabolism and which belong to the families 1–4 (4). However, 90% of drug oxidation seems to be related to six main enzymes: CYP 2D6, 2C9, 1A2, 2C19, 2E1, and 3A4 (5).

**Figure 1:**
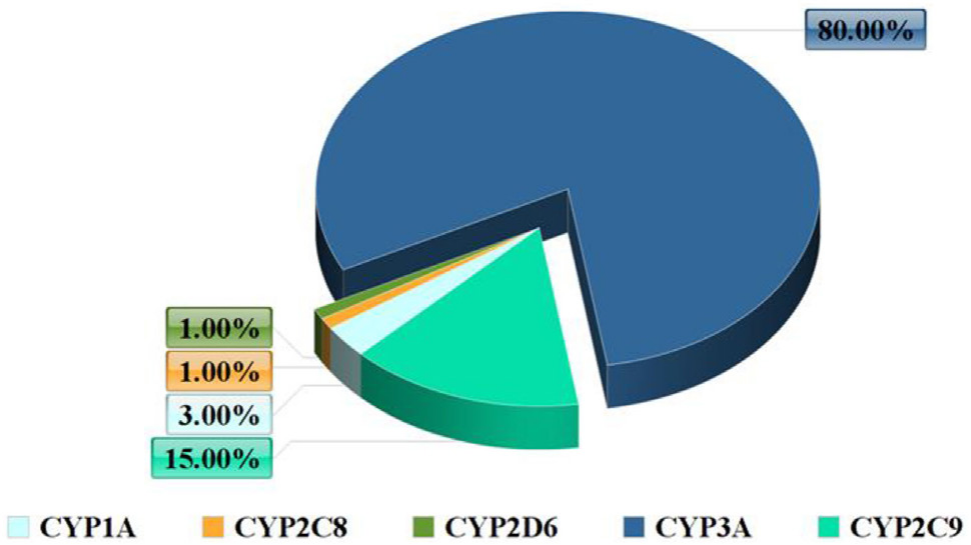
Frequency of the cytochrome P450 (CYP) system enzymes.

In terms of abundance, the most significant CYP enzymes are CYP3A4 and CYP2D6 (Figure 1). CYP3A4 is not expressed only in the liver but also in the intestinal tissue and it participates in the extrahepatic metabolism (6). The main role of the human intestinal tissue is the absorption of nutrients, although it also has the ability to metabolize drugs. CYP enzymes are in charge for the majority of phase I drug metabolism reactions.

**Figure 2:**
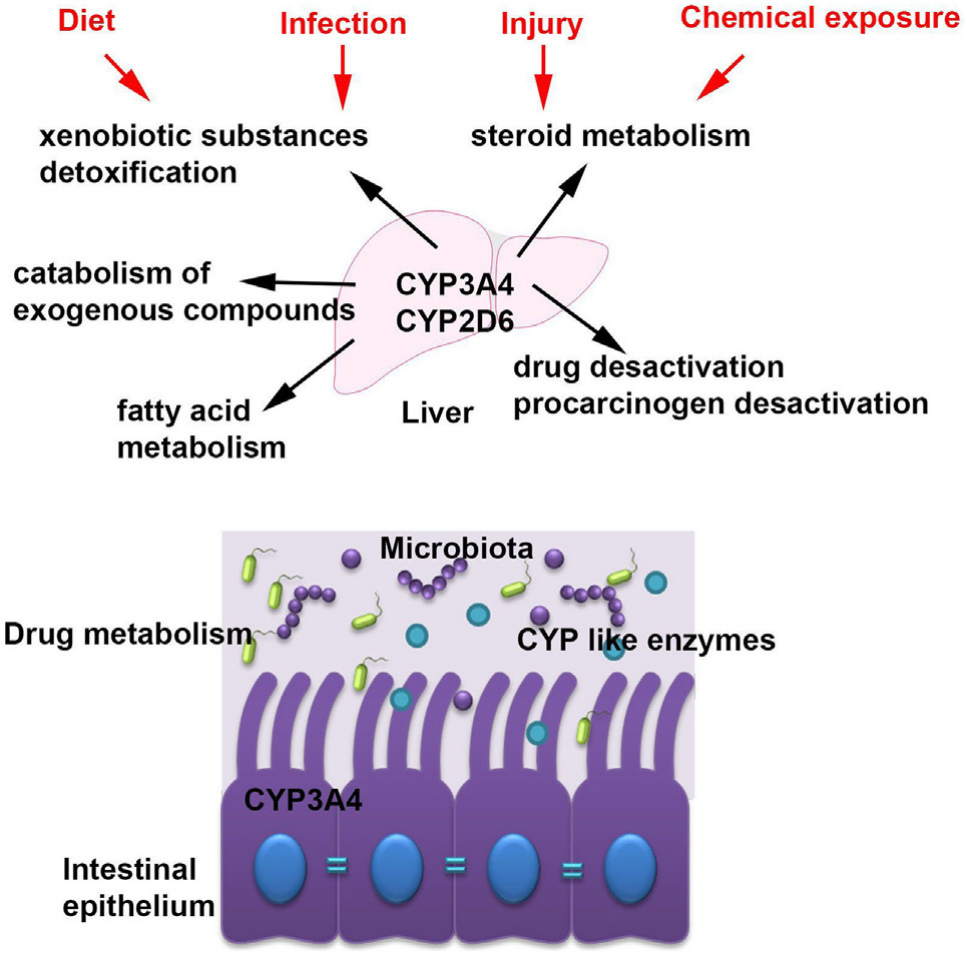
The main cytochrome P450 (CYP) enzymes and their role in host homeostasis. The most abundant CYP enzymes are CYP3A4 and CYP2D6 and they are expressed in the liver but also in the intestinal tissue. CYP enzymes are accomplish many functions within the host including steroid metabolism, drug and procarcinogen desactivation, fatty acid metabolism, xenobiotic substances detoxification, and catabolism of exogenous compounds and they are modulated by several factors such as diet, chemical exposures, infection, and injury.

Cytochromes are metabolizing a plethora of xenobiotic substances (2, 3) and are involved in many functions including steroid metabolism, drug and procarcinogen desactivation, fatty acid metabolism, xenobiotic substances detoxification, and catabolism of exogenous compounds (Figure 2) (2).

Cytochromes show intra- or interethnic and intra- or interindividual genetic variation. Differences in the pharmacokinetic status of the drug are related to the increased toxicity due to altered efficacy, reduced metabolism, elevated production of toxic metabolites, and adverse drug interactions (2, 4, 6).

In the intestine, the microbiota possess high-metabolic capacity due to its enormous pool of enzymes, which have the ability to catalyze reactions in phase I and phase II of drug metabolism (2). Cleavage of the intestinal barrier caused by the breakdown of gut integrity could increase passive paracellular absorption. It is evident that high- microbial abundance following aging, intestinal disturbances, environmental changes, or food-associated disease could determine the microbial metabolism of a drug before absorption. There is a possible multifactorial association of the CYP (P450) cytochrome role with different disease states, nutritional status, and environmental toxic effects (2). The hepatic CYPs have been associated with the pathogenesis of several liver diseases since CYP-mediated drug transformation into toxic metabolites may lead to hepatotoxicity. CYP3A4 is mostly expressed in the liver but it is also present in in the fetal liver and the brain, where it may play an important role in metabolism (6).

## In Light of the Inflammation Mechanism

Inflammation is the body response to stimuli such as pathogens, damaged cells, or toxins and consists of a protective response involving blood vessels, immune cells, and molecular mediators. The role of inflammation is to stamp out the source of cellular damage, eliminate necrotic cells, and injured tissues and to finally initiate tissue repair. Inflammation is characterized by four Latin words: *dolor, calor, rubor, tumor* (pain, heat, redness, and swelling). Inflammation is a general term, and it refers to the innate immunity components (cellular and molecular). Nevertheless, the adaptive immunity response can also activate and sustain the inflammatory response (e.g., complement system proteins) specific for each pathogen. Inflammation can be classified as acute or chronic and while prolonged inflammation, known as chronic inflammation may lead to a multitude of diseases, acute inflammation is the host immediate response to noxious stimuli and is attained by the migration of polymorphonuclear neutrophils from the blood into the damaged areas.

In parallel, several biochemical changes involving the immune system, the local vascular system, as well as various cells systems within the damaged tissue occur. It must be noted that inflammation is not synonymous with infection. Infection consists of the microbial invasion in an organism followed by the organism’s inflammatory defensive response. On the other hand, inflammation consists of the organism’s immune-vascular response, whatever the initial cause could be. In most cases of microbial invasive origin, there is a common background.

Inflammation is associated with an activation of the immune system characterized by release of cytokines, adipokines, lipid metabolites nitric oxide, proteases, and reactive oxygen species. The above inflammation mechanism comes along with the downregulation of the main xenobiotic/drug metabolizing CYP enzyme system (Figure 1) both in the liver and in the adipose tissue (7).

The innate immune system is stirred up by microbial invaders *via* pattern recognition receptors such as Toll-like receptor (TLR) or nucleotide-binding oligomerization-domain protein (NOD) families (8). Molecular sensors, named “inflammasomes” (cytoplasmic protein complexes harboring NOD-like receptor family proteins) could locate abnormalities in cellular components involved in tissue injury (9).

Inflammatory mediators include proinflammatory cytokines (i.e., IL-1β, IL-6, and TNFα), vasoactive amines (i.e., histamine), peptides (i.e., bradykinin), and lipid mediators (i.e., prostaglandins) (8). Stimulation of TLRs, NODs, or inflammasomes promotes the production of mediators that target for elimination of the microbial cells or are involved in the host tissue repairing processes (9, 10).

In response to inflammatory signals, the hepatic tissue shows increased synthesis and secretion of APP, α1-acid glycoprotein fibrinogen, and C-reactive protein, which are all involved in homeostasis (11).

It is obvious that infections can modulate pharmacokinetics of a drug *via* several mechanisms, which usually take place in the liver or the kidneys. The liver represents the key organ of metabolism clearance, and so disturbances in the expression or activities of drug metabolizing enzymes could modify hepatic clearance. There is also an important extrahepatic metabolism in the intestinal issue which is supported by a healthy intestinal system (2).

Inflammation or infection could cause important modifications in drug transporters and drug metabolizing enzymes in the liver or in intestinal epithelial cells thus resulting in a disturbance of oral bioavailability. Furthermore, there is a high specificity based on the fact that CYP enzymes can be regulated by a multitude of cytokines. Hence, CYP enzymes will be selectively regulated in different inflammatory states (11) and the pharmacokinetics could be affected following drug administration and infection.

When the inflammatory downregulation of CYP enzymes occurs at the different stages the reduction in P450 protein levels is preceded or accompanied by lower mRNAs levels, implicating transcription as a main mechanism. The transcription factor NF-κB is a key regulator of inflammation (12). Inflammatory stimuli can regulate the action of the pregnane X receptor (PXR) through binding the NF-κB p65 subunit to the retinoid X receptor (13, 14). Moreover, when NF-κB binds to the aryl hydrocarbon receptor, the occurring interaction results in a repression of their activity (15).

It should be mentioned that inflammation is associated with downregulations of hepatic and extrahepatic CYP enzymes drug metabolism. This could result in elevated plasma drug levels and adverse drug effects (3, 11).

## Experimental Data and Disease States

Acute adenovirus hepatitis in mice led to a selective downregulation of acetaminophen metabolizing CYP enzymes in liver (CYP 1A2 and CYP 2E1), reduced formation of acetaminophen toxic metabolites and thus lower risk of acetaminophen hepato-toxicity (16).

The CYP enzymes downregulation is dose, stimuli, and time dependent. Moreover, inhibition of drug metabolism as a result of interferon production and the stimulation of the cellular immunity was reported (17). Inflammation or administration of IL-6, IL-1, or TNF showed a decrease in the enzymatic activity of CYP 3A2, 1A1, 2C11, 2C12, and 2E1 (18).

Changes in CYP enzymes are acknowledged to be a frequent aftermath stimulation of the immune system following infection and inflammation (18–21) and in most cases there is no additional sign of toxicity of affected organs. CYP2C18 is expressed at very low levels in liver tissue, and so it is unaffected by cytokine exposure. The response pattern of cytochromes CYP2C9 and CYP2C19 was proven identical since both cytochromes were downregulated by TGF and IL-6 but not influenced by TNF, IL-1, IFN, or LPS (11). Moreover, data strongly suggest that even with the overlapping effects of cytokines, the activity of human P450s is independently regulated in infection and inflammation.

However, the differentiation in inflammatory response to cytokines may be crucial for patient treatment, since it is estimated that CYPs enzymes are distinctly regulated at different stages by various mechanisms, as a result to inflammation or disease. This foreknowledge is enhanced by the different sensitivity of CYP-dependent clearance revealed by infectious liver disease (22).

Cytochromes P450 enzymes are involved in the metabolism of a large pool of xenobiotic substances. Activation of cytochromes CYPs enzymes is influenced by a plethora of factors, such as genus, environment, disease state, alcohol consumption, and herbal medications (23).

In this frame, multiple isoforms of CYP have been linked to drug pharmacokinetics, carcinogenesis, steroids, and prostaglandins metabolism (23). As discussed previously, both P450 activity and level are affected by infection and inflammation, specifically by cytokines released during activation of the immune system.

Interferons and proinflammatory cytokines can downregulate P450 expression *ex vivo* (in hepatocyte cultures) and *in vivo*, and these modulators are believed to be the reason of P450 downregulation in the inflammatory process (24).

Drug–drug interactions can occur during anticancer therapy with interferon or ILs. Moreover, in patients with secondary hepatic cancer, treated with IL-2, dose-dependent reductions in CYP1A2, CYP2E1, CYP2C, and CYP3A4 protein levels were observed (25).

Cytochromes P450 enzymes are activated by specific substances and agents (23). In animal models, the effects of inflammatory mediators on the expression of CYPs (3) following IL-6, IL-1, or TNF administration caused a decline in enzyme activity for CYPs 2C11, 2C12, 1A1, 2E1, and 3A2. The decrease in catalytic activity was associated in most cases with a proportional decrease in protein and in mRNA levels (3). CYP 4A2, 4A1, and 4A3 liver expression was elevated after LPS treatment of rats (26).

The ability of LPS and BaSO_4_ to suppress the expression of several cytochromes like CYP 4F4 and CYP 4F5 seems qualitatively equal albeit the response to LPS stimulation is quantitatively higher. It is then conceivable that the operational mechanism for LPS and BaSO_4_ may be distinct (27). The downregulation of CYPs enzymes in the LPS animal model of inflammation seems to be mediated by oxidative stress, since an antioxidant regimen inhibits the downregulation of CYP 1A1, 3A11, and 2E1 and PXR (28–30).

As discussed, infections or inflammation cause alterations in the expression levels and activities of various forms of P450 in the liver, as well as in other extrahepatic tissues such as kidney and brain (31). However, in most cases, the activity of CYPs is inhibited.

It must be also noted that the effects of inflammatory mediators are not limited only to infections with live organisms or inflammatory ailments: interferons are used in cancer treatment and antiviral therapy, and various other cytokines are under *in vivo* investigation for cancer treatment (3, 31).

CYP2E1 is activated in brain astrocytes during the inflammatory process and CYP1A1 is downregulated (32), whereas intracerebroventricular injection of bacterial LPS suppresses CYP1A (33). Moreover, injection of LPS in the brain has produced important effects on the liver activity of CYP 3A, CYP1A, CYP2B, and CYP2E1 (34).

It must be highlighted that the modulation of CYP P450 by inflammatory cytokines was extensively studied in cultured rat hepatocytes (31). Moreover, studies performed to detect the effects of cytokines on cultured human hepatocytes (35–37) showed effects identical to those observed in rat hepatocytes (31). However, it was reported that important levels of CYP2A6 were found in postmortem specimens of liver infected with hepatitis B or C virus in infected cells or cells found in areas proximity of fibrosis or inflammation (31) harbored. Conversely, the hepatitis A virus was connected to a decreased CYP2A6 enzyme clearance (38).

Inflammation is associated to chronic diseases, as a result to the tissue’s adaptation to a cellular stress or dysfunction (3, 8) which is usually less abrupt than an acute response to infection or tissue damage. In patients with advanced cancer the downregulation is linked to CYP3A4 (39) and in animal model systems is mediated *via* IL-6 derived from the tumor itself (9).

Increased CYP3A4 and CYP3A5 mRNA expression levels were also detected in pediatric Crohn’s disease patients (40). In the light of the above, it is clear that more research is needed to clarify these conflicting results and the effects of the different infectious or inflammatory diseases on the expression and activities of different human P450 enzymes. Thus, patients under a stable drug dosage who are infected would develop increased exposure to the drug *via* a reduced clearance and/or increased bioavailability, and so then adverse effects should be developed (3, 9). This was the case when an influenza epidemic lead to a reduced clearance of theophylline in children taking asthma medication (41).

Vaccination and immune stimulation seem to play a critical role. Drug metabolism is compromised in humans with impaired immune system after inflammation or vaccination (42). Toxicity could be induced due to enhanced pharmacological responses as a result of the downregulation of drug metabolizing enzymes by the cytochromes, in the patients with infections or following vaccination or in cancer patients receiving interferon or in patients under cytokine therapy (42). It must also be reported that patients receiving anticonvulsants or theophylline, which require systematic monitoring of their serum levels, often seem to have an impaired drug metabolism after vaccination (41).

Influenza virus vaccination downregulate CYP 1A2 enzymes expression (43). In immunodeficiency virus-positive patients CYP 2D6 enzymes expression was also decreased (44). Cytokines linked to effector T-cell responses can alter the regulation of many drug transporters as well as CYP enzymes levels. Immune-modulating antibodies used in cancer therapy may have effects on CYP enzymes (45). It seems that there is a close connection between obesity, immunity, and inflammation (46, 47), as many CYP enzymes are expressed in the adipose tissue (48, 49).

Obese hospitalized patients seem to develop more frequently endonosocomial infections. Mortality of obese patients with severe sepsis was higher compared with non-obese patients (48, 49). Genetic predisposition seems to be a critical factor.

Chronic, heavy alcohol exposure contributes to major pathophysiological effects associated to ethanol and inflammation of the adipose tissue, insulin resistance, and liver injury. Moreover, ethanol feeding increased CYP2E1 expression in adipocytes (50).

Aged mice whose retina was exposed briefly to 670 nm light, which increases mitochondrial membrane potential and reduces inflammation showed significant increases in levels of cytochrome *c* oxidase, which is a mitochondrial enzyme modulating oxidative phosphorylation (51).

It was also reported that the phagocytic activity and secretory capacity of Kupffer cells is closely associated with increased immune reactions and downregulated expression of some hepatic cytochrome CYP (52). In the same time, the inhibition of Kupffer cell by GdCl3 (gadolinium chloride) exerted anti-obesity effects in high-fat diet-fed mice (52). All these data show that obese individuals are not only more susceptible to infections, but also have a greater risk for adverse drug reactions due to impaired drug metabolism and kinetics (52).

To date, there is not much information available to discriminate between the expression of CYPs in acute versus chronic inflammation. The expression of different CYP isoenzymes was studied by Muntane et al. using the Carrageenan-induced granuloma which permits the separation between acute and chronic phases of experimental inflammation (53). CYP2D, CYP2E1, CYP3A1, and CYP4A were reduced to 20% of the control level during the acute phase of inflammation, and partially recovered (that is 30–60% of control group) during the chronic phase of inflammation (53). CYP2B1 expression was decreased to 65% of control during the acute and chronic phases of inflammation. CYP2B1 and CYP2E1 revealed a strong depression during the acute phase of inflammation and recovered during the chronic phase (53).

## Conclusion

The CYP represent a superfamily of enzymes with a key role in the activation or inactivation of a plethora of therapeutic agents. CYP enzymes are involved in the metabolism of xenobiotic substances. Cytochromes present intra- or interindividual and intra- or interethnic genetic polymorphisms. Variations in the pharmacokinetic drug profile are linked to the rising toxicity following a declining metabolism, reduced efficacy of the drug, adverse drug interaction, and increasing production of toxic metabolites. The high-metabolic rate of the intestinal microbiota is due to its many enzymes which catalyze reactions in phase I and II drug metabolism. In case of a compromised intestinal barrier, there may be an increase in paracellular passive absorption.

It is evident that high-microbial abundance following intestinal disturbances, environment, aging, or food-associated diseases promotes the microbial metabolism of a drug before absorption. Recently, the beneficial effect of certain microbes on the intestinal ecosystem has been largely discussed. The aim of probiotics is to restore the deficiencies in the intestinal microbiota and establish a protective effect. There is a multifactorial association of the CYP enzyme role in the different disease states, nutritional status, and environmental toxic effects. CYP cytochromes keep a key role in cancer formation and cancer treatment as they activate numerous precarcinogens and participate in the inactivation and activation of anticancer drugs (54). However, the question is raised, which specific variant of the CYP alleles should be related to the different type of cancers? In general, CYPs enzymes involved in precarinogen’s activation are not polymorphic, in contrast to the CYPs enzymes which enter into the drug biotransformation where large interindividual differences are noted in the drug metabolizing ability for therapeutic purposes due to polymorphic alleles (55).

As discussed extensively, many of the CYP are modulated by injury and infection. Knowledge of the enzyme specificity and of the regulatory mechanisms will allow to figure out effectively the drug dosage regimens during a patient’s inflammatory status.

It is known that cytokines are useful markers in certain inflammatory diseases. Albeit, IL-6 deletion seems to weaken the downregulation of certain CYP enzymes in mice under inflammatory stimuli regimen. It is then conceivable that cytokines cannot be the unique biomarker of an inflammatory disease. Acute phase proteins, as well as other metabolomics and proteomic markers need to be determined in order to reveal the infectious status and confirm diagnosis (56).

## Author Contributions

ES wrote the manuscript, GP made figures, corrected and submitted the manuscript, EB drafted the manuscript.

## Conflict of Interest Statement

The authors declare that the research was conducted in the absence of any commercial or financial relationships that could be construed as a potential conflict of interest. The reviewer VL declared a past co-authorship with one of the authors (EB) to the handling editor.
